# A Case of Acute Osteomyelitis: An Update on Diagnosis and Treatment

**DOI:** 10.3390/ijerph13060539

**Published:** 2016-05-27

**Authors:** Elena Chiappini, Greta Mastrangelo, Simone Lazzeri

**Affiliations:** 1 Infectious Disease Unit, Meyer University Hospital, University of Florence, Florence 50100, Italy; greta.mastrangelo@gmail.com; 2 Orthopedics and Traumatology, Meyer University Hospital, Florence 50100, Italy; simone.lazzeri@meyer.it

**Keywords:** acute hematogenous osteomyelitis, children, bone infection, infection biomarkers, osteomyelitis treatment

## Abstract

Osteomyelitis in children is a serious disease in children requiring early diagnosis and treatment to minimize the risk of sequelae. Therefore, it is of primary importance to recognize the signs and symptoms at the onset and to properly use the available diagnostic tools. It is important to maintain a high index of suspicion and be aware of the evolving epidemiology and of the emergence of antibiotic resistant and aggressive strains requiring careful monitoring and targeted therapy. Hereby we present an instructive case and review the literature data on diagnosis and treatment.

## 1. Case Presentation

**Figure 1:**
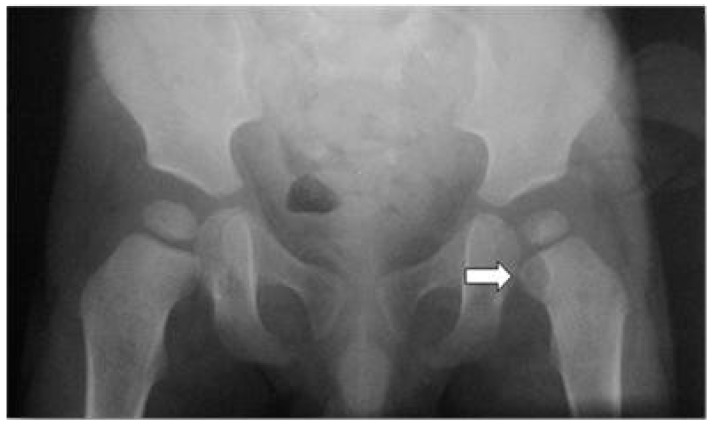
X-rays of the pelvis. Osteolytic lesion of the proximal metaphysis of the left femur (arrow).

A previously healthy 18-month-old boy presented at the emergency department with left hip pain and a limp following a minor trauma. His mother reported that he had presented fever for three days, cough and rhinitis about 15 days before the trauma, and had been treated with ibuprofen for 7 days (10 mg/kg dose every 8 h, orally) by his physician. The child presented with a limited and painful range of motion of the left hip and could not bear weight on that side. Examination of the other joints was unremarkable, and no inflammatory signs were evidenced. Blood tests were performed and showed white blood cell (WBC): 23,000 cells/µL; neutrophils: 86%, C-reactive protein (CRP): 7.2 mg/dL; erythrocyte sedimentation rate (ESR): 52 mm/h. Blood cultures were taken at admission, before administration of antibiotic therapy, and yielded negative results. Ultrasound scan of the hip was normal. X-rays of the pelvis and left hip showed a lytic lesion of the proximal femoral metaphysis (Figure 1).

**Figure 2:**
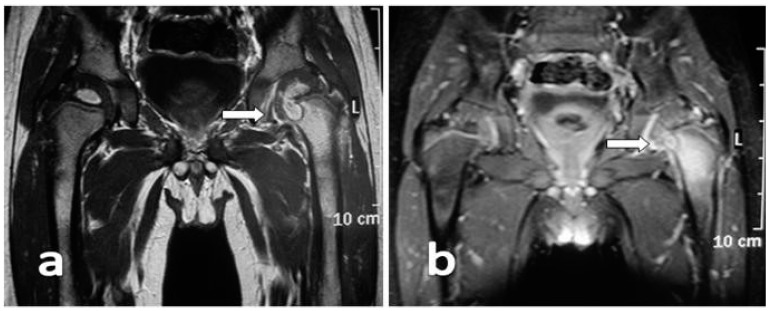
MRI confirms osteomyelitis of the femoral neck with a possible involvement of the growth plate without involvement of the joint cavity (arrow). (**a**) T1-weighted image showing metaphyseal fluid collection with surrounding edema; (**b**) T1-weighted SPIR image enhancing the fluid component.

The child was admitted for suspected acute osteomyelitis of the proximal femur and a Magnetic Resonance Imaging (MRI) was ordered. The gadolinium-enhanced MRI confirmed the clinical suspicion of osteomyelitis (Figure 2). After orthopedic consultation, a closed needle biopsy, and drainage of the lesion was performed. Specimens were was sent for histological and microbiological examination and empiric intravenous (IV) antibiotic treatment with ceftazidime and oxacillin was started. Specimen cultures grew methicillin-sensitive *Staphylococcus aureus* (MSSA). The child showed rapid clinical improvement, and normalization of inflammatory markers.

After 7 days, intravenous therapy was stopped and replaced with oral flucloxacillin (25 gm/kg every 6 h) for an additional 4 weeks. Six months after discharge the child showed clinical improvement and reached pain-free full range of motion of his left hip. Gait was normal, as well as blood tests, and X-rays findings.

## 2. Introduction

Osteomyelitis is an infection of bone sustained most commonly by bacteria, although fungal etiology is rarely described, particularly in immunocompromised children [1]. According to the time period between diagnosis and symptom onset, osteomyelitis is classified as acute (<2 weeks), sub-acute (2 weeks–3 months), or chronic (>3 months). Bacteria may reach bone marrow through the bloodstream, or spreading from nearby tissue. Infection can also be subsequent to an injury that exposes bone to a contaminated environment [1].

The estimated incidence of acute osteomyelitis is about 8 cases per 100,000 children/year [2,3] Children under 5 years of age are affected in about 50% of the cases, with a M:F ratio of 2:1. Acute osteomyelitis is approximately two times more frequent than septic arthritis, and its incidence is steadily increasing. Gafur *et al.* [4] observed that the incidence of acute osteomyelitis has tripled over the last 20 years while the incidence of septic arthritis remained constant. Early detection is crucial given that a delay in the diagnosis of only 4 days is a risk factor for long-term sequelae (Table 1).

Possible complications include septic arthritis, subperiosteal abscess, pyomyositis, deep vein thrombosis, sepsis, and multiorgan failure. Even if the mortality is less than 1%, permanent disabilities can occur, such as growth arrest with limb length discrepancy. Moreover, acute osteomyelitis can evolve to the chronic form.

Most of pediatric osteomyelitis originates as a bloodstream infection. The route of entry may be the respiratory tract, particularly for *K. kingae*, *S. pyogenes* and *S. pneumoniae*, while the skin may be a common port of entry for *S. aureus* [3]. Less frequently, acute osteomyelitis spreads from contiguous tissues or from direct inoculation following trauma or surgery.

## 3. Diagnosis

The case described is instructive in many ways: medical history, clinical findings and treatment.

Medical history is typical, since the child had both a previous upper airway infection and a previous trauma, which may be an additional risk factor for acute osteomyelitis [5,6]. During bacteremia, pathogens may reach the metaphyseal regions of bones via the nutrient arteries and their straight branches, connecting with venous sinusoids near the physis. Blood flow in this area is slow and turbulent, and bacterial may enter into the extravascular space, after passing through endothelium gaps in the metaphyseal vessels [7]. A peculiar anatomic feature occurs in children aged less than 18 months: transphyseal blood vessels exist only in this age group and may allow bacteria to reach the epiphysis from the metaphysis [7,8]. Thus, in young children, osteomyelitis originating in the metaphyseal region can spread to the epiphysis, with destruction of both.

The location of the infection in our patient is typical as well. Primary hematogenous osteomyelitis most frequently involves the metaphysis of long bones, *i.e.*, femur (23%–29%), tibia (19%–26%), and humerus (5%–13%). The lower extremity is more commonly affected. Multifocal forms are uncommon, and usually associated with sepsis, and occur in about 5%–7% of pediatric cases, mostly in newborns (22%) [1,3].

In the described case of osteomyelitis, the patient presented with pain, functional limitation, and elevation of inflammatory markers, but without fever. Indeed, it should be kept in mind that the absence of fever does not rule out osteomyelitis, and that the classic triad of fever, pain and increased markers of inflammation is not always present. Dartnell *et al.* [9] reported that fever was the presenting symptom only in 61.7% of children with acute osteomyelitis, while pain and swelling and erythema where present in 81.1% and 70% of the cases, respectively. Reduced mobility is present in about 50% of children with osteomyelitis and frequently young children may present with little more than a limp or refusal to weight bear. Extreme functional impairment with an almost complete reduction of range of motion in a joint suggests septic arthritis rather than osteomyelitis, where tenderness over a metaphyseal region of a long bone is more common.

In a recent study, leukocytosis has been reported only in 36% of children, increased ESR (cutoff value 20 mm/h) in 91%, and elevated CRP value (cutoff value 20 mg/dL) in 81% of them [10]. The sensitivity of ESR and CRP reached 98% when both were increased. Procalcitonin (PCT) has been investigated as biomarker for bacterial infection with mixed results [11,12,13]. In a prospective study evaluating WBC, ESR, PCR and PCT, the latter one was found to be the most sensitive (85.2%) and specific (87.3%) test to differentiate osteomyelitis from non-infectious diseases [14]. The authors proposed a PCT cut off >0.4 ng/mL as a threshold to initiate treatment for acute osteomyelitis. Nevertheless, the role of PCT as biomarker for osteoarticular infections is still under investigation. In a recent study including over 500 patients, the sensitivity of PCT was only 67%, while the specificity reached 90% [15]. Blood cultures are positive in about 50% of the paediatric cases [16], while bone aspirates may give positive results in 70% of the cases.

In a limping child the differential diagnosis should include traumatic, rheumatologic, and neoplastic diseases (Table 2). In a child with musculoskeletal pain the probability of cancer is 1: 10,000 and a pediatric Gait, Arms, Legs, Spine screening (pGALS) [17] examination may help identify red flags that raise concern about infection or malignancy. In a recent retrospective study [18] including 286 children with acute lymphoblastic leukemia, 20% had a joint-localized bone pain as presenting symptom and 17% of them were initially erroneously diagnosed to have osteomyelitis.

X-ray studies in the presented case showed an osteolytic lesion of the proximal metaphysis of the femur. Indeed, in the acute phase of the disease, X-ray sensitivity is low (43%–75%), while specificity is slightly higher (75%–83%) [19]. X-rays can be normal up to 14 days after the onset of symptoms, and even after two weeks, only 20% of the cases show radiographic changes. The lytic lesions are evident only when at least 50% of bone is destroyed. Nevertheless, performing an initial X-ray is important to rule out alternative diagnoses (*i.e.*, fractures or tumors). Ultrasound scan is usually negative (sensitivity 46%–74%, specificity 63%–100%) and therefore has a limited use. It is useful to recognize fluid collections in joints and soft tissues and to guide a possible biopsy [20].

MRI (preferably with paramagnetic contrast medium) is the main investigation tool, having both high sensitivity (82%–100%), and specificity (75%–99%). It is useful to localize the lesion, to define its extension, to follow up the development of the disease and to plan for surgical intervention [19]. Differential diagnosis between Ewing’s sarcoma and AOM maybe challenging. X-ray findings of both osteomyelitis and Ewing sarcoma are often similar with an aggressive intramedullary process destroying normal cancellous and cortical bone creating a moth eaten and permeative appearance. MRI can be helpful in the differential diagnosis. Several features, including periostitis, periosteal elevation, adjacent soft tissue mass and effacement of fat planes are common to the two diseases, as well as soft tissue enhancement, cystic and necrotic foci and cortical destruction. Fat globules inclusions within the marrow process or in the subperiostium are suggestive of acute osteomyelitis more often than cancer. In a recent study including 35 subjects permeative cortical involvement and soft-tissue mass were more likely in subjects with Ewing’s sarcoma, whereas a serpiginous tract was more likely to be seen in subjects with osteomyelitis [21].

It should be remembered that in young children RMI requires anesthesia. Bone scan using 99Tc has been proven to be sensitive and useful in some circumstances, especially poor clinical localization or multiple foci of infection Morevoer, it may help to distinguish ongoing infection to reparative activity and to monitor response to treatment, it is associated with small doses of irradiation, and does not require anesthesia [22].

**Figure 3:**
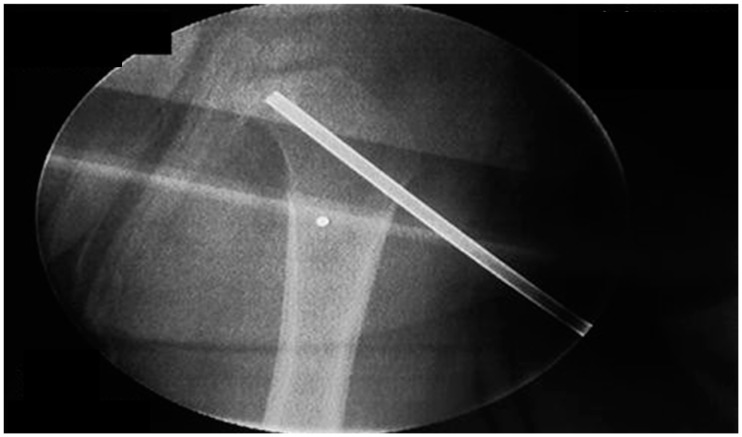
Image intensifier control during surgical closed needle biopsy and drainage.

In the presented case, needle biopsy and debridement of the lesion was performed (Figure 3). Cultures of specimen obtained after surgical drainage may grow the pathogen in up to 70% of the cases. Biopsy is recommended to secure diagnosis, considering that several conditions, especially Ewing’s sarcoma, frequently mimic acute osteomyelitis and a delay of the actual diagnosis would be detrimental. On the other hand, surgical treatment of acute osteomyelitis in children is not routinely recommended [23].

The most common causative infectious organisms are *Staphylococcus aureus* (70%–90% of positive cultures) followed by *Streptococcus pyogenes*, *Streptococcus* p*neumoniae* and Gram negative bacilli. Streptococci and Gram negative bacilli cause up to 60% of infections in children under the age of 4 [16]. For this reason, a combined antibiotic therapy, covering Gram positive and Gram negative strains, (ceftazidime plus oxacillin) was chosen in our case. However it should be underlined that the increased prevalence of Gram negative organisms is also due to *K. kingae* infection, and the incidence is increasing predominantly due to improved detection methods, including polymerase chain reaction. Over the last 10 years, the pattern of pathogens involved has changed (Table 3). On one hand, causative pathogens such as *Haemophilus influenzae* type *B*, once the most common Gram negative organism involved in paediatric osteomyelitis, have become rare as a consequence of the vaccination campaigns. On the other hand, cases associated with *Kingella kingae* infection are rising as are methicillin-resistant *S. aureus* (MRSA) cases, albeit with wide geographical variations. In fact, MRSA is responsible for up to 9%–30% of osteomyelitis in children [24,25,26]. In our regions data are poor. Local epidemiological data indicate low proportion of MRSA isolated from community acquired osteomyelitis (<10%) in Italy, supporting the use of oxacillin.

Osteomyelitis due to MRSA is usually more aggressive, with higher inflammation markers, prolonged hospital stays and increased possibility to undergo surgical treatment. Therefore, these forms are associated with a higher likelihood of developing complications such as deep vein thrombosis, pulmonary embolism, multifocal infections, subperiosteal abscesses formation, multiorgan failure and progression to chronic osteomyelitis. The association with these complications is more frequent in the event of Panton-Valentine Leukocidin (PVL)-producing isolates. Geographical differences have been reported. In the U.S. the predominant PVL-producing *S. aureus* strains are MRSA [27]. On the other hand, in Europe, PVL-producing *S. aureus* strains are more commonly MSSA which have been associated with severe osteoarticular infections in children [28,29]. Several studies have demonstrated PVL production is stimulated by subinhibitory concentrations of betalactams [29], which is the case in abscesses, therefore clindamycin or other antibiotic drugs inhibiting protein synthesis are recommended especially in the presence of pulmonary infections by these strains.

## 4. Update on Therapeutic Management

Caring for children with acute haematogenous osteomyelitis is a multidisciplinary challenge and requires collaboration between pediatricians, infectious diseases specialists, orthopedic surgeons, microbiologists and radiologists. Copley *et al.* [25] confirmed the effectiveness of a multidisciplinary approach in terms of efficiency of clinical investigations, rates of identifications of causative pathogens and length of hospital stays. The main goal of this common effort is to establish an early and effective antibiotic therapy. 

The choice of a specific antibiotic is based on the identification of the causative infectious organism and on local epidemiological data on resistance. An antistaphylococcal penicillin such as oxacillin or flucoxacillin and/or a cephalosporin are recommended as first line treatment. Some authors [1] have suggested to use antibiotics affective against MRSA while awaiting for culture results, especially in settings with more than 10% of *S. aureus* isolates are MRSA or if risk factors are present. This approach is, however, not generally accepted since it is thought that this may contribute to the spread of antibiotic resistant strains. In a recent review [24] different regimens depending on MRSA local prevalence where proposed (Table 4). The duration and routes of administration of antibiotics is currently under debate [30]. Historically, osteomyelitis was treated with intravenous antibiotics for 4–6 weeks. In the only randomized trial [31] that has addressed the issue of the duration of therapy, patients who showed a good clinical response after 2–4 days of intravenous treatment and where shifted to oral treatment for further 20 days, had the same outcome of children treated with continued IV therapy for 30 days. This approach has been adopted in many centers, although it is usually patient tailored, depending on the organism being treated, local bacterial sensitivity epidemiological data, availability of oral equivalent antibiotic, and severity of the osteomyelitis [32]. However, the generalization of the results of the only available trial [31] study is debatable. It has been highlighted that the study population is unique and, indeed, that MSSA had been isolated in almost 90% of cases, showing a peculiar epidemiology.

More recently, Keren *et al.* [32] retrospectively evaluated data from 2060 children with osteomyelitis: 1005 received a brief IV antibiotic treatment (7 days), followed by oral therapy and 1055 received IV therapy for 4–6 weeks. Similar treatment failure rates were observed (approximately 5%), while almost 15% of children receiving prolonged IV therapy developed deep vein thrombosis. These results support the trend to rapidly shift from IV to oral therapy, at least in children that show a good clinical response in terms of resolution of fever, pain and restoration of function, combined with progressive normalization of inflammatory markers. On the other hand, it has to be acknowledged that, given the retrospective observational nature of the study, criticism to this approach exists.

Faust *et al.* [33] noted that the two treatment groups in the study by Keren [32] were not perfectly homogeneous: the children treated with oral antibiotics were more frequently younger than one year of age and in worse socio-economic conditions. Others [34] pointed out that many data regarding the severity of the disease (*i.e.*, duration of symptoms before admission, duration of fever, trend of inflammatory markers) were lacking.

Tamma *et al.* [35] speculated that children on prolonged IV therapy had more severe disease and emphasized the lack of information about the discrepancies between antibiotic used and susceptibility of the isolated pathogen. Pending the results of other clinical studies, a reasonable approach could be to perform a short (5–7 days) IV therapy with a subsequent shift to oral administration in uncomplicated cases, in children over 3 months of age. Prolonged treatment (at least 14–21 days, to be decided case by case) could be reserved to children under 3 months of age, to complicated cases (such as multifocal osteomyelitis), to immunocompromised patients, and to patients at high risk of complications (*i.e.*, sickle cell anemia, sepsis, extensive destruction of bone, isolation or high suspicion of antibiotic resistant microorganism).

In some cases, transition to oral therapy can be difficult in the clinical setting, since the limited availability of oral antibiotics. The dosage of oral antibiotic therapy is under debate as well. In a recent review published by Peltola *et al.* [24], same doses as per parenteral route are recommended for oral treatment. Faust *et al.* [33], on the other hand, recommend much lower oral doses. Moreover, the successful use of trimethoprim/sulfamethoxazole (TMP/SMX) for oral treatment of MRSA acute osteomyelitis has been reported from the U.S. [36]. At our institution we switch to oral therapy after 5–7 days if course is favorable, using the highest recommended doses. In proven or suspected infections by MRSA, we consider different therapeutic options for the switch to oral treatment, including also a combination therapy with TMP/SMX plus rifampicin.

## 5. Conclusions

Acute osteomyelitis in children is a serious disease that, when detected and treated early, can heal without severe sequelae [37]. It is of primary importance to recognize the signs and symptoms at the onset of the disease and to properly use the available diagnostic tools [38,39]. The role of serum markers as predictive factors for diagnosis has not been completely established. In particular, PCT should be further evaluated in larger studies and a cut-off value has not been univocally defined. Similarly, recommendations for the duration of intravenous antimicrobial therapy have not been stated. Moreover, local different prevalence of antibiotic resistant strains may justify different therapeutic approaches. Of note, the widely adopted short course antibiotic therapy has not been investigated in poor resource countries, where the timing of diagnosis is delayed and other comorbidities such as anemia, malnutrition and HIV infection may influence the outcomes. The authors’ opinion is that, at the moment, every child with acute osteomyelitis should receive a “tailored therapy”, based on epidemiological resistance data, age, initial response to first intravenous antibiotic regimen, availability of oral drugs for the suspected causative infectious agent, potential compliance of the family to oral therapy after discharge, risk of adverse events and costs.

## Figures and Tables

**Table 1:** Risk factors for long term sequelae.

Risk Factors for Long-Term Sequelae
Late diagnosis (>4 days)
Inadequate treatment
Neonate (prematurity, hypoxia, central venous catheterization)
Sickle cell disease
Infection by MRSA or Panton-Valentine Leukocidin positive strains

**Table 2:** Differential diagnosis.

Differential Diagnoses
Reactive arthritis
Juvenile arthritis
Septic arthritis
Trauma
Cancer (osteoid osteoma, leukemia, eosinophilic granuloma, metastatic neuroblastoma, Ewing’s sarcoma, osteosarcoma)

**Table 3:** Age-specific etiologic agents of pediatric acute osteomyelitis.

Age Group	Common Pathogens
0–3 months	*Staphylococcus aureus*
	*Streptococcus agalactiae*
	Gram negative enteric bacteria
3 months–4 years	*Staphylococcus aureus*
	*Streptococcus pyogenes*
	*Kingella kingae*
	*Haemophilus influenzae* type b (in non-immunized child)
>5 years	*Staphylococcus aureus*
	*Streptococcus pyogenes*

**Table 4:** Proposed antibiotic treatment for acute osteomyelitis in children [24].

Bacteriology	Antibiotic	Dose mg/kg/die	Maximum Daily Dose	Bone Penetration ^#^
If MRSA prevalence in the community <10%	First generation cephalosporin *	150 divided into 4 equal doses	2–4 g	6–7
	**OR**			
	Antistaphylococcal penicillin (cloxacillin, flucloxacilina, dicloxacillin, nafcillin, or oxacillin)	200 divided into 4 equal doses	8–12 g	15–17
If the prevalence of MRSA in the community >10% and the Prevalence of *S. aureus* resistant to clindamycin <10%	Clindamycin	40 divided into 4 equal doses	3 g	65–78
If the prevalence of MRSA in community ≥10% and the Prevalence of *S. aureus* clindamycin resistente ≥10%	Vancomycin	40 divided into 4 equal doses Or 45 mg divided in 3 equal doses	Dose adjusted according to blood levels with a target of 15–20 μg/mL trough level	5–67
	**OR**			
	Linezolid if vancomycin is not effective	30 divided in 3 equal doses	1.2 g no more than 28 days	40–51
Alternatives for specific agents	Ampicillin or amoxicillin for Beta-hemolytic streptococcus group A, *Haemophilus influenzae* type b (strains which do not produce beta-lactamase, *S. pneumoniae* sensitive to penicillin	150–200 dispensed in 4 equal doses	8–12 g	3–31

* Cephalotin and cefazolin iv, cephalexin and cefadroxil per os, cephadrine both iv and per os. If active first generation cephalosporins are not available, cefuroxime may be used iv. ^#^ As bone concentration/blood concentration ratio (%).

## Abbreviations

The following abbreviations are used in this manuscript:

WBC	White Blood Cell
CRP	C-reactive protein
ESR	Erythrocyte Sedimentation Rate
MRI	Magnetic Resonance Imaging
MSSA	Methicillin-sensitive *Staphylococcus aureus*
MRSA	Methicillin-resistant *Staphylococcus aureus*
PCT	Procalcitonin
pGALS	pediatric Gait, Arms, Legs, Spine
TMP/SMX	trimethoprim/sulfamethoxazole
